# Sociodemographic, Clinical, and Ventilatory Factors Influencing COVID-19 Mortality in the ICU of a Hospital in Colombia

**DOI:** 10.3390/healthcare12222294

**Published:** 2024-11-16

**Authors:** Claudia Lorena Perlaza, Freiser Eceomo Cruz Mosquera, Sandra Patricia Moreno Reyes, Sandra Marcela Tovar Salazar, Andrés Fernando Cruz Rojas, Juan Daniel España Serna, Yamil Liscano

**Affiliations:** Grupo de Investigación en Salud Integral (GISI), Departamento Facultad de Salud, Universidad Santiago de Cali, Cali 5183000, Colombia; patyreyes01@gmail.com (S.P.M.R.); marceemarr@gmail.com (S.M.T.S.); andresfernandocruz1995@gmail.com (A.F.C.R.); espanaserna@gmail.com (J.D.E.S.); yamil.liscano00@usc.edu.co (Y.L.)

**Keywords:** COVID-19, SARS-CoV-2, risk factors, sociodemographic factors, mortality, mechanical ventilation, critical care

## Abstract

Background and Objectives: The COVID-19 pandemic posed significant challenges to healthcare systems worldwide, and mortality rates were driven by a complex interaction of patient-specific factors, one of the most important being those related to the scheduling of invasive mechanical ventilation. This study examined the sociodemographic, clinical, and ventilatory factors associated with mortality in COVID-19 patients admitted to the ICU of a hospital in Colombia. Methods: A retrospective cohort study was conducted, involving 116 patients over the age of 18 who were admitted to the ICU with a confirmed diagnosis of COVID-19 between March 2020 and May 2021. Data were collected from the patients’ medical records. Statistical analysis was performed using SPSS version 24^®^. Odds ratios (OR) and 95% confidence intervals were calculated to identify factors associated with COVID-19 mortality, followed by adjustment through binary logistic regression. Results: It was found that 65.5% of the patients were male, with a mean age of 64 ± 14 years, and the overall mortality rate was 49%. Factors significantly associated with higher mortality included male sex (OR: 6.9, 95% CI: 1.5–31.7), low oxygen saturation on admission (OR: 7.6, 95% CI: 1.1–55), and PEEP settings at 96 h (OR: 8, 95% CI: 1.4–45). Mortality was not influenced by socioeconomic status or health system affiliation. Conclusions: This study identified male sex, age over 65 years, PEEP greater than 10 cmH_2_O at 96 h of mechanical ventilation, and low oxygen saturation as significant factors associated with higher mortality in COVID-19 patients, while no significant associations were found with socioeconomic status or health system affiliation. These findings highlight the importance of focusing on clinical management and ventilatory strategies in reducing mortality, particularly for high-risk groups, rather than relying on socioeconomic factors as predictors of outcomes.

## 1. Introduction

Coronavirus disease (COVID-19), caused by the SARS-CoV-2 virus, was first identified in Wuhan, China, in late 2019. By January 2020, Chinese authorities officially declared SARS-CoV-2 as the causative agent of this rapidly spreading infection [1]. The virus quickly triggered a surge of cases and deaths in China, which soon escalated into a global public health crisis [2]. According to the World Health Organization (WHO), by 2022, more than 500 million confirmed cases and over six million deaths were reported worldwide [3,4,5,6].

During the pandemic, the Chinese Center for Disease Control and Prevention highlighted that individuals at higher risk of severe COVID-19 complications were those with pre-existing medical conditions and inadequate sanitary conditions [7]. In China, studies showed that between 5% and 32% of patients admitted to emergency departments required transfer to intensive care units (ICU) for further management [8].

Research has consistently linked ICU admissions and COVID-19 mortality to underlying metabolic disorders, cardiovascular diseases, and other non-communicable chronic conditions [9,10,11,12,13]. Additionally, factors such as advanced age, chronic illnesses like hypertension, diabetes, cardiovascular disease, obesity, and smoking were found to significantly worsen COVID-19 outcomes [7,8,9].

In Latin America, in addition to the previously described factors, various studies have assessed the influence of socioeconomic disparities on the outcomes of patients with COVID-19. A study conducted in Mexico by Antonio et al. [14], reported excess mortality among individuals living in marginalized and densely populated communities. They also found that cases treated in public health institutions had a lower likelihood of hospitalization and a higher risk of death. Meanwhile, research in Chile revealed that cities with higher residential overcrowding experienced a greater excess mortality due to COVID-19 [15]. In Brazil, Marques et al. [16] reported an increased risk of death among men, older adults, and residents in areas with poorer social conditions.

Colombia reported 865 confirmed COVID-19 cases by January 2020, with 8% requiring ICU admission. However, few studies have explored the sociodemographic, clinical, and ventilatory factors associated with COVID-19 mortality in Colombia, especially in cities where social and cultural dynamics affect health outcomes. The city of Cali had the second-highest COVID-19 mortality rate in Colombia in 2022 (44.5 per 100,000 inhabitants), exceeding the global average. This difference may be related to a high prevalence of chronic diseases, limited access to healthcare services, hospital capacity, socioeconomic inequalities, slow vaccination rates, and a high population density [17,18].

Several studies have pointed to sociodemographic and economic factors as key determinants of clinical outcomes [19,20]. However, in the Colombian context, the relationship between socioeconomic status, healthcare access, and mortality has not been clearly established. This study, therefore, aims to identify the sociodemographic, clinical, and ventilatory factors influencing COVID-19 mortality in ICU patients in Cali, Colombia. Given the significant challenges posed by ICU mortality, particularly among vulnerable populations, it is essential to deepen the understanding of these contributing factors.

## 2. Materials and Methods

### 2.1. Type of Study

A retrospective cohort study was conducted on 116 subjects over 18 years of age with a molecular diagnosis of COVID-19, admitted to an intensive care unit (ICU) in Cali, Colombia, between March 2020 and May 2021. Fourteen patients (*n* = 14) were excluded due to insufficient information in their medical records for analysis.

### 2.2. Sociodemographic Variables

Sociodemographic variables considered in the study included sex (male/female), occupation, marital status, age (in years), health system affiliation, and socioeconomic status. According to the National Administrative Department of Statistics of Colombia (DANE), socioeconomic status was classified based on the location of the individual’s residence, with higher strata indicating better economic status [21]. For this study, socioeconomic status was dichotomized as low (strata 1 to 3) or high (strata 4 to 6). Additionally, ethnic self-identification was included, considering the groups established by DANE, such as Afro-descendants, Indigenous peoples, Mestizos, and Romani. This variable was considered due to documented social and health disparities among different ethnic groups in the country. In particular, communities such as Afro-Colombians and Indigenous peoples face significant barriers in accessing healthcare services at various levels, which could translate to a higher risk of death during the COVID-19 pandemic [22].

### 2.3. Clinical Variables

Clinical variables included respiratory rate, heart rate, oxygen saturation, Fraction of Inspired Oxygen (FiO_2_), blood pressure, and body mass index (BMI), calculated using the patient’s weight and height. Additional variables associated with organ dysfunction were also considered, such as Acute Respiratory Distress Syndrome (ARDS), liver failure, kidney failure, arrhythmias, septic shock, and any relevant medical history.

### 2.4. Ventilatory Variables

Ventilatory variables include the initial tidal volume (Vt), (FiO_2_), initial respiratory rate, initial positive end-expiratory pressure (PEEP), PEEP at 48 h, and PEEP at 96 h. These variables were selected due to the influence that mechanical ventilation settings, particularly oxygenation parameters, have on COVID-19 patient outcomes during the first four days of hospitalization, as shown in previous studies [23,24]. Additionally, the days from hospital admission to the start of invasive mechanical ventilation (IMV), days from ICU admission to the start of IMV, total days on IMV, and whether the patient was placed in the prone position were considered.

### 2.5. Paraclinical Variables

Paraclinical variables evaluated in the study included arterial blood gas values upon ICU admission, basic hematology, and biochemistry. The primary outcome variable was the patient’s discharge status, which was dichotomized as either alive or deceased.

### 2.6. Data Collection

Data collection was conducted by three healthcare professionals who extracted information from the institution’s electronic medical records. The data were then entered into a Microsoft Excel^®^ database (accessed on 20 August 2021), which had been pre-constructed by the researchers based on the variables of interest.

### 2.7. Statistical Analysis

The data were analyzed using SPSS^®^ version 24 (accessed on 20 August 2021). Initially, the normality of the distribution of quantitative variables was assessed using the Kolmogorov–Smirnov test with a Lilliefors correction. Categorical variables were expressed as frequencies and percentages, while quantitative variables were presented as means ± standard deviations. Differences in proportions were analyzed using the Chi-square test or Fisher’s test, and differences in means were assessed using the Student’s *t*-test or the Mann–Whitney U test, depending on data normality. A *p*-value of <0.05 was considered statistically significant.

To determine the association between independent variables and ICU mortality, crude odds ratios (ORs) were calculated. Finally, a binary logistic regression model was performed, using the Hosmer–Lemeshow goodness-of-fit test, to adjust for variables with a *p*-value < 0.20 in the univariate analysis. The results of the logistic regression model were presented in a table that reported the exponent B along with its corresponding 95% confidence interval.

### 2.8. Ethical Considerations

This study was conducted in accordance with the Declaration of Helsinki and Resolution 008430 of 1993, issued by the Ministry of Health and Social Protection of Colombia. The research was approved by the ethics committee of Universidad Santiago de Cali under Act No. 03 CEB. All participant data, obtained from medical records, were treated confidentially and were accessible only to the researchers. Informed consent was not required due to the retrospective nature of the study, which involved the review of existing clinical data. Additionally, obtaining consent from patients, many of whom may have passed away, was not feasible. The healthcare institution granted approval, and all data were handled in compliance with national regulations to ensure the protection of participant identities.

## 3. Results

### 3.1. Patient Demographics

The studied cohort had an average age of 64 ± 14 years, with the majority being male (65.5%), predominantly of Mestizo ethnicity (78%), followed by Afro-descendants (17%), and all participants were classified within socioeconomic strata 1 to 3 (100%) (see Table S1).

### 3.2. Mortality Rate

Of the studied sample, 57 patients died during their stay in the intensive care unit (ICU), resulting in a mortality rate of 49%. Upon evaluating the sociodemographic and clinical characteristics at hospital admission, statistically significant differences were observed in terms of sex and the service through which patients were admitted to the health institution. Additionally, patients who died exhibited a greater tendency towards oxygen desaturation compared to survivors (deceased: 82 ± 16 vs. survivors: 88 ± 9, *p* = 0.01) (see Table 1).

### 3.3. Clinical Characteristics at ICU Admission

Regarding the clinical characteristics at ICU admission, patients who died showed a slight tendency towards tachycardia compared to survivors (91 ± 19 vs. 84 ± 17, *p* = 0.039). Gasometric analysis revealed significant differences between groups in pH levels (survivors: 7.41 ± 0.08 vs. deceased: 7.35 ± 0.15, *p* = 0.028) and serum bicarbonate levels (survivors: 24 ± 5 vs. deceased: 21 ± 4, *p* = 0.003). It is noteworthy that the frequency of requiring non-invasive respiratory support (low or high flow oxygen therapy) upon ICU admission was similar between survivors and non-survivors; moreover, no subjects were placed on non-invasive mechanical ventilation (see Table S2).

### 3.4. Ventilatory Support

In comparing variables related to invasive mechanical ventilation (IMV) within the study population, it was evident that deceased patients required IMV more frequently than survivors (93% vs. 46%, *p* = 0.0001). Additionally, deceased patients more frequently developed acute respiratory distress syndrome (ARDS) (58% vs. 37%, *p* = 0.026) and septic shock (61% vs. 37%, *p* = 0.009). Regarding ventilator settings, patients who died had higher levels of PEEP at 96 h and a slightly higher respiratory rate (see Table 2).

### 3.5. Blood Chemistry

In terms of blood chemistry, deceased patients had significantly higher white blood cell counts compared to survivors (13.6 ± 6.5 vs. 10.9 ± 4.4, *p* = 0.011), as well as slightly higher hemoglobin levels. Additionally, a tendency towards hyperchloremia was observed in deceased patients compared to survivors (109 ± 8.5 vs. 105 ± 6.7, *p* = 0.006), and higher levels of lactate dehydrogenase were recorded (818 ± 483 vs. 660 ± 319, *p* = 0.041) (see Table S3).

### 3.6. Logistic Regression Analysis

Binary logistic regression revealed that the model was statistically significant (*p* = 0.001) and explained 48% of the variability in the outcome (Nagelkerke R^2^ = 48%). Independent factors associated with mortality in COVID-19 ICU patients included male sex (OR: 6.9, 95% CI: 1.5–31.7), low oxygen saturation at admission (OR: 7.6, 95% CI: 1.1–55), PEEP set at 96 h (OR: 8, 95% CI: 1.4–45), and age over 65 years (OR: 20.5, 95% CI: 3.3–126) (see Table 3).

## 4. Discussion

### 4.1. Main Findings

During the COVID-19 pandemic, healthcare professionals working in critical care areas faced the immense challenge of caring for severely ill patients [25]. Despite their dedicated efforts, COVID-19 significantly impacted mortality rates within ICUs, as evidenced by this study, which reported a mortality rate of 49% among the patients examined. This finding aligns with previous studies that have reported ICU mortality rates ranging from 45% to 65% [26,27,28]. Variations in COVID-19 mortality rates in ICUs can be attributed to several factors. Primarily, the increasing understanding of the disease facilitated more timely interventions, thereby improving clinical outcomes. Additionally, differences in sociodemographic characteristics and access to medical resources play a crucial role in the progression of critically ill patients, directly influencing mortality rates [29].

In this study, age over 65 years and male sex were independently associated with higher mortality, a result supported by earlier research [30,31]. The increased risk of COVID-19 mortality in older adults is attributed to age-related factors, such as immunosenescence, a high prevalence of comorbidities, and a greater propensity for exaggerated inflammatory responses, which increase the likelihood of tissue damage and multiorgan failure. Additionally, the progressive decline in lung function and generalized frailty limit their ability to recover and tolerate intensive interventions, significantly heightening their vulnerability to infection [32]. Furthermore, previous studies suggest that male sex is a risk factor for mortality due to a higher expression of the angiotensin-converting enzyme 2 (ACE2), which facilitates viral entry and increases viral load. This is compounded by the coexistence of comorbidities and behavioral risk factors, such as smoking and delayed medical attention, which escalate disease severity and the associated complications [33].

Patients admitted with a low oxygen saturation and those with a PEEP > 10 cmH_2_O at 96 h had a significantly higher risk of death. These findings are explained by the direct impact of these variables on ventilatory mechanics and oxygenation status, indicating a more severe clinical state and a higher risk of mortality [34]. However, no significant relationship was found between socioeconomic status or health system affiliation and mortality, likely because the studied population shared similar vulnerability characteristics.

This is consistent with studies in Latin America, where the relationship between socioeconomic status and mortality is not always direct. Factors such as the structure of the health system and access to services can moderate the impact of socioeconomic status on clinical outcomes. Similarly, in countries like Brazil, which were severely affected by COVID-19, comorbidities and age were found to be more determinant factors in mortality than economic inequalities [35].

Studies conducted in Colombia suggest that COVID-19 mortality related to socioeconomic inequalities depends on the responsiveness of local health systems. In some cases, early and adequate responses to the pandemic may have mitigated the impact of socioeconomic status and other aspects, such as health system affiliation, on mortality. However, this position remains controversial [18]. Among the aspects that may have influenced the lack of a relationship between socioeconomic factors and mortality in this study are limitations in the detailed collection of such data and the single-center design of the research. This restricts the ability to generalize findings to other populations with different socioeconomic conditions and access to healthcare. Additionally, the absence of comprehensive information on key variables, such as the availability of medical resources and the timeliness and quality of medical care, may have affected the interpretation of their impact on COVID-19 mortality, as these factors are not always systematically recorded in retrospective studies.

It is worth noting that the R^2^ value of the model (48%) suggests that it only explains part of the variability in COVID-19 mortality. This indicates the presence of unconsidered factors, such as the presence of comorbidities (cardiovascular, metabolic, or respiratory diseases), disease severity at admission, and healthcare-related factors (timeliness, access, and quality of care). Including these factors could improve the model’s explanatory power and provide a more comprehensive understanding of the determinants of mortality in COVID-19 patients.

Finally, we highlight that, although this aspect was not included in the model, 59% of survivors were directly admitted to the ICU compared to 37% of non-survivors, suggesting that early admission and intensive intervention may have contributed to better outcomes in surviving patients. This finding underscores the importance of rapid critical care and proactive management in severe COVID-19 cases and highlights the need for further studies to explore how ICU admission timing impacts patient outcomes.

### 4.2. Clinical Implications

Clinical management of critically ill COVID-19 patients requires targeted strategies to optimize outcomes, particularly in patients at high risk of mortality, such as older males with severe hypoxemia and elevated PEEP requirements. Evidence suggests that oxygenation targets should be carefully managed, with recent trials, including the HOT-ICU trial, indicating that aiming for a lower partial pressure of arterial oxygen (PaO_2_) of around 60 mmHg (8 kPa) can result in more days alive without life support compared to higher targets around 90 mmHg (12 kPa) [36,37,38]. For patients on mechanical ventilation, adhering to lung-protective strategies, such as using low tidal volumes (4–8 mL/kg of predicted body weight) and maintaining plateau pressures (Pplat) ≤ 30 cm H_2_O, is crucial to minimize ventilator-induced lung injury (VILI). Additionally, the optimization of PEEP levels should strike a balance between oxygenation and lung protection, avoiding excessively high pressures that could worsen lung injury [39].

In cases where patients exhibit refractory hypoxemia despite optimal ventilatory management, adjunctive therapies, such as prone positioning and neuromuscular blockade, should be considered, as they have been shown to improve oxygenation and outcomes in severe ARDS cases. Early intervention and continuous monitoring of high-risk patients, especially older males with low oxygen saturation, are essential, with driving pressure (ΔP) serving as a useful parameter to guide ventilatory adjustments [40]. Furthermore, advanced technologies, such as machine learning models, can assist in predicting individualized oxygenation targets, helping to tailor oxygenation strategies to patient-specific characteristics, potentially reducing mortality [40]. A comprehensive approach to care, which includes addressing comorbid conditions and providing supportive care like fluid management and nutritional support, remains critical in managing these patients [41,42]. Implementing these evidence-based strategies can help mitigate identified risk factors and improve survival rates among ICU COVID-19 patients.

In summary, the findings of this study highlight the importance of precise monitoring and adjustment of PEEP levels to prevent ventilator-induced lung injury and ensure adequate oxygenation in critically ill COVID-19 patients. Early correction of hypoxemia through ventilation adjustments and oxygen therapy techniques is essential to prevent the progression to severe respiratory failure [37]. Additionally, continuous monitoring of arterial blood gases and respiratory mechanics, accompanied by a personalized ventilation strategy, are key interventions to optimize management and improve clinical outcomes in this patient group [40].

### 4.3. Limitations and Strengths

The limitations of this study are related to the use of medical records as the data source, which may have affected the uniformity in the measurement of certain variables. Additionally, some relevant clinical variables, such as D-dimer levels, were not included, despite being identified as potential mortality predictors in other studies [43]. Additionally, it is important to note that certain confounding factors, such as prevalent comorbidities (cardiovascular diseases, obesity, diabetes, and chronic respiratory diseases), were not considered in the analysis. These conditions, that are well established as risk factors, significantly influence COVID-19 clinical outcomes by increasing disease severity, altering therapeutic responses, and predisposing patients to complications [34]. Given that this study was conducted in a single hospital, the results may not be entirely representative of other populations or clinical settings, which could limit the generalizability of the findings. Multicenter studies would be useful to assess their applicability in broader contexts. Another potential limitation of this study is the combination of variables measured at admission and during ICU stay in the model, which may have introduced an immortality bias [44]. This bias could have influenced the results, and future studies are recommended to consider the appropriate timing for including these variables to minimize their impact.

Finally, in-depth social variables that could explain socioeconomic inequalities and social vulnerability in this population were not thoroughly explored. However, an important strength of this study is that it is one of the few to investigate factors associated with mortality in a hospital that became a reference center for the care of vulnerable patients during the pandemic.

## 5. Conclusions

This study found that male sex, age over 65 years, PEEP greater than 10 cmH_2_O at 96 h of mechanical ventilation, and low oxygen saturation levels were strongly associated with increased mortality. However, no significant associations were identified with low socioeconomic level (stratum 1 to 3) or health insurance scheme, indicating that clinical factors play a more decisive role in mortality outcomes than socioeconomic conditions, in the sample studied.

## Figures and Tables

**Table 1:** Comparison of sociodemographic variables and clinical characteristics at hospital admission between survivors and non-survivors (*n* = 116).

Variable	Survivors (*n* = 59) *n* (%)	Non-Survivors (*n* = 57) *n* (%)	Total (*n* = 116) *n* (%)	*p*-Value
Sex				
Male	34 (58%)	42 (74%)	76 (66%)	0.059
Female	25 (42%)	15 (26%)	40 (34%)	
Health Insurance Scheme				
Subsidized	34 (58%)	40 (70%)	74 (64%)	
Contributory	20 (34%)	12 (21%)	32 (27%)	0.310
Beneficiary	4 (7%)	5 (9%)	9 (8%)	
Special	1 (1%)	0 (0%)	1 (1%)	
Admission Service				
Outpatient Consultation	12 (19%)	5 (9%)	17 (14%)	
Emergency Room	1 (1%)	11 (19%)	12 (10%)	0.001
ICU	34 (59%)	21 (37%)	55 (48%)	
Intermediate Care Unit (UCIM)	12 (21%)	20 (35%)	32 (28%)	
Days Between Symptom Onset and Hospital Admission	8.3 ± 8.5	8.1 ± 7.9	8.2 ± 8.0	0.839
Glasgow Coma Scale at Admission	14.6 ± 0.8	14 ± 1	14 ± 1.5	0.100
Clinical Symptoms at Admission				
Chest Pain				
Yes	5 (9%)	7 (12%)	12 (10%)	0.501
No	54 (91%)	50 (88%)	104 (90%)	
Shortness of Breath				
Yes	43 (73%)	42 (74%)	85 (73%)	0.922
No	16 (27%)	15 (26%)	31 (27%)	
Anosmia				
Yes	7 (12%)	3 (5%)	10 (9%)	0.205
No	52 (88%)	54 (95%)	106 (91%)	
Asthenia				
Yes	33 (56%)	38 (67%)	71 (61%)	0.236
No	26 (44%)	19 (33%)	45 (39%)	
Cough				
Yes	39 (66%)	38 (68%)	77 (66%)	0.949
No	20 (34%)	19 (32%)	39 (34%)	
Temperature (°C)	36.4 ± 0.6	36.4 ± 0.7	36.4 ± 0.7	1.000
Oxygen Saturation (SaO_2_)	88 ± 9	82 ± 16	85 ± 13	0.015
Pre-ICU Oxygen Support				
Yes	51 (86%)	49 (86%)	100 (87%)	0.941
No	8 (14%)	8 (14%)	16 (7%)	
Transfer from Another Hospital				
Yes	34 (58%)	32 (56%)	66 (57%)	0.872
No	25 (42%)	25 (44%)	50 (43%)	

Abbreviations: ICU: Intensive Care Unit; UCIM: Intermediate Care Unit; SaO_2_: Oxygen Saturation.

**Table 2:** Comparison of variables related to invasive mechanical ventilation in the studied patients (*n* = 116).

Variable	Survivors (*n* = 59) *n* (%)	Non-Survivors (*n* = 57) *n* (%)	*p*-Value
Invasive Mechanical Ventilation			0.0001
Yes	27 (46%)	53 (93%)	
No	32 (54%)	4 (7%)	
Ventilatory Parameters			
Initial Vt (mL)	357 ± 44	363 ± 51	0.500
Initial PEEP (cmH_2_O)	10.5 ± 1.7	10 ± 1.8	0.127
PEEP at 48 h (cmH_2_O)	10.1 ± 1.6	10.4 ± 1.8	0.345
PEEP at 96 h (cmH_2_O)	9.2 ± 2	10.1 ± 1.8	0.012
Initial FiO_2_ (%)	79 ± 25	83 ± 20	0.343
Initial Respiratory Rate (breaths/min)	16 ± 2	17 ± 3	0.038
Time Variables			
Days Between Hospital Admission and Start of IMV	3.9 ± 3.5	4 ± 4	0.886
Days Between ICU Admission and Start of IMV	2.4 ± 2.2	2.1 ± 1.7	0.412
Days on IMV	8 ± 9	8.7 ± 7.5	0.649
ARDS			0.026
Yes	22 (37%)	33 (58%)	
No	37 (63%)	24 (42%)	
Prone Position			0.139
Yes	24 (41%)	31 (54%)	
No	35 (59%)	26 (46%)	
Septic Shock			0.009
Yes	22 (37%)	35 (61%)	
No	37 (63%)	22 (39%)	

Abbreviations: Vt: Tidal Volume; PEEP: Positive End-Expiratory Pressure; FiO_2_: Fraction of Inspired Oxygen; IMV: Invasive Mechanical Ventilation; ARDS: Acute Respiratory Distress Syndrome.

**Table 3:** Factors associated with COVID-19 mortality in the study population (*n* = 116).

Variables	Standard Error	OR	Wald	95% CI (Lower–Upper)	*p*-Value
Male Sex	0.775	6.9	6.2	1.5–31.7	0.012
Low Oxygen Saturation on Admission	1.010	7.6	4.0	1.1–55.5	0.044
PEEP > 10 cmH_2_O at 96 h	0.886	8.0	5.5	1.4–45.5	0.019
ARDS	0.743	3.0	2.2	0.7–13.1	0.133
Septic Shock	0.877	1.1	0.0	0.2–6.3	0.879
Age > 65 Years	0.925	20.5	10.6	3.3–126.1	0.001

Abbreviations: PEEP: Positive End-Expiratory Pressure; ARDS: Acute Respiratory Distress Syndrome.

## Data Availability

Data are contained within the manuscript.

## Supplementary Materials

The following supporting information can be downloaded at https://www.mdpi.com/article/10.3390/healthcare12222294/s1, Table S1: Sociodemographic characteristics of the studied population (*n* = 116); Table S2: Comparison of clinical characteristics at ICU admission between survivors and non-survivors included in the study (*n* = 116); Table S3: Comparison of paraclinical results between survivors and non-survivors included in the study (*n* = 116).
