# Supplementary material for: Sociodemographic, Clinical, and Ventilatory Factors Influencing COVID-19 Mortality in the ICU of a Hospital in Colombia
Source: Healthcare (Basel). 2024 Nov 16;12(22):2294. doi: 10.3390/healthcare12222294 (PMC11593780; doi:10.3390/healthcare12222294)
Supplement: Supplementary file 1 [file healthcare-12-02294-s001.zip › healthcare-3295510-supplementary.pdf]

Supplementary Materials

Table S1. Sociodemographic characteristics of the studied population (n=116).

Variable	Frequency (n)	(%)
**Sex**		
Male	76	65.5
Female	40	34.5
**Ethnicity**		
Mestizo	90	78
Afro-descendant	20	17
Indigenous	6	5
**Health Insurance Scheme (SGSSS)**		
Subsidized	74	63.8
Contributory	32	27.6
Beneficiary	9	7.8
Special	1	0.9
**Socioeconomic Stratum**		
1— 3	116	100
4— 6	0	0
**Mean age**	64	14*
**Mode age**	63	19-94**

Abbreviations: SGSSS: General System of Social Security in Health; *Standard Deviation;** Range.

Table S2. Comparison of clinical characteristics at ICU admission between survivors and non-survivors included in the study (n=116)

Variable	Survivors (n=59) n(%)	Non-Survivors (n=57) n(%)	*p*-value
**Non-invasive respiratory support (NIRS) at ICU admission**			
Yes	53 (91%)	50 (89%)	0.719
No	6 (9%)	7 (11%)	

**Non-invasive mechanical ventilation (NIMV)**	0 (0%)	0 (0%)	--
**Respiratory rate** (breaths/min)	25 ± 5	27 ± 7	0.081
**Oxygen saturation (SaO_2)** (%)	89 ± 8	85 ± 11	0.028
**Heart rate** (beats/min)	84 ± 17	91 ± 19	0.039
**Systolic blood pressure** (mmHg)	130 ± 30	127 ± 32	0.604
**Diastolic blood pressure** (mmHg)	75 ± 13	73 ± 19	0.511
**Mean arterial pressure** (mmHg)	93 ± 7	91 ± 34	0.665
**Glasgow score**	14 ± 1	14 ± 1	1.000
**Arterial blood gases**			
Ph	7.41 ± 0.08	7.35 ± 0.15	0.028
$PaCO_2$ (mmHg)	37 ± 13	38 ± 11	0.655
HCO_3 (mEq/L)	24 ± 5	21 ± 4	0.003
PaO_2 (mmHg)	76 ± 27	81 ± 32	0.366
SaO_2 (%)	92 ± 5	91 ± 6	0.332
PaO_2/FiO_2	140 ± 81	144 ± 88	0.792

Abbreviations: NIRS: Non-invasive respiratory support; NIMV: Non-invasive mechanical ventilation; SaO_2: Oxygen saturation; $PaCO_2$: Partial pressure of carbon dioxide; HCO_3: Bicarbonate; PaO_2: Partial pressure of oxygen; FiO_2: Fraction of inspired oxygen.

Table S3. Comparison of paraclinical results between survivors and non-survivors included in the study (n=116)

**Variable**	**Survivors (n=59)**	**Non-Survivors (n=57)**	***p*-value**
**White blood cell count** (x10^3)	10.9 ± 4.4	13.6 ± 6.5	0.011
**Neutrophil count** (x10^3)	78.1 ± 9.0	90.7 ± 86	0.265
**Eosinophil count** (x10^3)	1.07 ± 0.85	1.02 ± 0.88	0.756
**Lymphocyte count** (x10^3)	13.7 ± 7.2	13.2 ± 15.5	0.824
**Monocyte count** (x10^3)	6.0 ± 3.6	5.8 ± 3.4	0.759
**Hemoglobin** (g/dL)	13 ± 1.8	14.8 ± 1.8	0.001
**Platelets** (x10^3)	304 ± 123	276 ± 120	0.217
**Sodium (Na)** (mEq/L)	137 ± 5.1	161 ± 180.2	0.309
**Potassium (K)** (mEq/L)	4.1 ± 0.6	4.2 ± 0.8	0.447
**Chloride (Cl)** (mEq/L)	105 ± 6.7	109 ± 8.5	0.006
**Lactate** (mg/dL)	20 ± 11	25 ± 23	0.136
**C-reactive protein** (mg/dL)	79.4 ± 44	83.8 ± 34.9	0.516
**LDH** (UI/L)	660 ± 319	818 ± 483	0.041
**Ferritin** (ng/mL)	942 ± 481	1064 ± 541	0.203

Abbreviations: Na: Sodium; K: Potassium; Cl: Chloride; LDH: Lactate dehydrogenase.
